# Challenges with scale-up of GeneXpert MTB/RIF® in Uganda: a health systems perspective

**DOI:** 10.1186/s12913-020-4997-x

**Published:** 2020-03-04

**Authors:** Talemwa Nalugwa, Priya B. Shete, Mariam Nantale, Katherine Farr, Christopher Ojok, Emma Ochom, Frank Mugabe, Moses Joloba, David W. Dowdy, David A. J. Moore, J. Lucian Davis, Adithya Cattamanchi, Achilles Katamba

**Affiliations:** 1 Department of Medicine, Makerere University College of Health Sciences, Kampala, Uganda; 2 Uganda Tuberculosis Implementation Research Consortium, Kampala, Uganda; 3 Division of Pulmonary and Critical Care Medicine, University of California San Francisco and Zuckerberg San Francisco General Hospital 5K1, San Francisco, CA 94110 USA; 4 Uganda National Tuberculosis and Leprosy Control Programme, Kampala, Uganda; 5 School of Biomedical Sciences, Makerere University College of Health Sciences, Kampala, Uganda; 6 Department of Epidemiology, Johns Hopkins Bloomberg School of Public Health, Baltimore, MD USA; 7 Faculty of Infectious and Tropical Diseases and TB Centre, London School of Hygiene and Tropical Medicine, London, UK; 8 Department of Epidemiology of Microbial Diseases, Yale School of Public Health, New Haven, CT USA; 9 Section of Pulmonary, Critical Care, and Sleep Medicine, Yale School of Medicine, New Haven, CT USA

**Keywords:** Tuberculosis, Health system, Infrastructure, GeneXpert, Infectious disease, Diagnosis

## Abstract

**Background:**

Many high burden countries are scaling-up GeneXpert® MTB/RIF (Xpert) testing for tuberculosis (TB) using a hub-and-spoke model. However, the effect of scale up on reducing TB has been limited. We sought to characterize variation in implementation of referral-based Xpert TB testing across Uganda, and to identify health system factors that may enhance or prevent high-quality implementation of Xpert testing services.

**Methods:**

We conducted a cross-sectional study triangulating quantitative and qualitative data sources at 23 community health centers linked to one of 15 Xpert testing sites between November 2016 and May 2017 to assess health systems infrastructure for hub-and-spoke Xpert testing. Data sources included a standardized site assessment survey, routine TB notification data, and field notes from site visits.

**Results:**

Challenges with Xpert implementation occurred at every step of the diagnostic evaluation process, leading to low overall uptake of testing. Of 2192 patients eligible for TB testing, only 574 (26%) who initiated testing were referred for Xpert testing. Of those, 54 (9.4%) were Xpert confirmed positive just under half initiated treatment within 14 days (*n* = 25, 46%). Gaps in required infrastructure at 23 community health centers to support the hub-and-spoke system included lack of refrigeration (*n* = 14, 61%) for sputum testing and lack of telephone/mobile communication (*n* = 21, 91%). Motorcycle riders responsible for transporting sputum to Xpert sites operated variable with trips once, twice, or three times a week at 10 (43%), nine (39%) and four (17%) health centers, respectively. Staff recorded Xpert results in the TB laboratory register at only one health center and called patients with positive results at only two health centers. Of the 15 Xpert testing sites, five (33%) had at least one non-functioning module. The median number of tests per day was 3.57 (IQR 2.06–4.54), and 10 (67%) sites had error/invalid rates > 5%.

**Conclusions:**

Although Xpert devices are now widely distributed throughout Uganda, health system factors across the continuum from test referral to results reporting and treatment initiation preclude effective implementation of Xpert testing for patients presenting to peripheral health centers. Support for scale up of innovative technologies should include support for communication, coordination and health systems integration.

## Background

The global tuberculosis (TB) epidemic continues with an estimated 10.0 million cases in 2017, of which an estimated 3.7 million “missing” cases were either undiagnosed or unreported to public health authorities [1]. To improve the diagnostic cascade of care for patients, there has been significant global and country level investment in rapid and more sensitive diagnostic tests such as GeneXpert MTB/RIF® (Xpert) (Cepheid, Sunnyvale, USA). Since the World Health Organization (WHO) endorsed Xpert in 2010, there has been massive scale-up of Xpert worldwide. As of 2016, 6659 Xpert instruments (containing approximately 30,000 testing modules) and over 23 million cartridges have been procured through concessional pricing available to low-income countries in order to reduce the burden [2].

Because of the substantial cost and infrastructure requirements of 4-module Xpert deployment, many high burden countries have adopted a “hub-and-spoke” model for scale-up. In this model, four module Xpert devices are placed at higher-level health facilities with adequate infrastructure requirements including security, stable power supply (hubs), each of which receive sputum samples from several lower level health facilities (spokes). The goal of these hub-and-spoke units is to expand coverage of Xpert testing services using existing devices and infrastructure, thereby increasing access to rapid and more sensitive diagnostic testing for patients who present to lower level health facilities.

Although Xpert is undoubtedly a pivotal innovation for TB diagnostics in high burden settings [3], the impact of Xpert scale-up on improvement of TB outcomes has been negligible. A recent systematic review and meta-analysis of Xpert use in programmatic settings demonstrated no benefit in morbidity or mortality for individual patients [4]. Many health systems issues have been hypothesized to explain this lack of effect: from poor infrastructure to limited quality systems for maintaining Xpert devices; to lack of funding and use of empiric treatment. However, to date no studies have assessed health system barriers to Xpert implementation at the facility level [5]. We sought to characterize variation in implementation of referral-based Xpert TB testing across Uganda, and to identify health system factors that may reduce or enhance efficient implementation of Xpert testing services.

## Methods

### Study setting and context

Uganda first adopted the use of Xpert for TB diagnosis in patients living with HIV or requiring testing for possible multidrug-resistant (MDR) TB in 2011. In September 2017, Uganda adopted policy recommendations in line with WHO guidelines that recommends Xpert testing where available for all patients with possible TB [6]. With support from partners, Uganda has installed 249 Xpert devices in 227 of 1500 (15%) TB diagnostic units in the country [6]. To date, Xpert devices have been deployed at all regional or district hospitals, most of which are serving as testing hubs. Local motorcycle (i.e.*,* boda) riders have been employed by Central Public Health Laboratories (CPHL) with support from implementing partners to carry sputum specimens from community health centers to Xpert testing hubs and to return to the community health centers with results. With the establishment of this hub and spoke network, the Ministry of Health intended that all patients with signs or symptoms of TB presenting to community health facilities with TB diagnostic units would have access to rapid, referral-based Xpert testing services. Each hub serves approximately 20–40 community health centers, and each rider’s daily route is expected to cover 4–8 facilities (NTRL, personal communication).

Community health centers were included in the study if they: 1) used sputum smear microscopy as the primary method for TB diagnosis; 2) participated in external quality assurance testing for sputum smear microscopy led by the National Tuberculosis and Leprosy Program (NTLP); and 3) were linked to an Xpert testing site/hub. Community health centers were excluded if they: 1) performed sputum smear examination on < 150 patients per year based on 2015 NTLP data; 2) diagnosed < 15 smear-positive TB cases per year; or 3) were located within Kampala District or > 150 km from central Kampala City. Of 1105 level III-IV community health facilities in Uganda that reported to the NTLP, 27 met eligibility criteria based on review of 2015 NTLP TB testing and treatment data. Of those eligible sites, 23 were selected for the study after consultation with the NTLP. TB testing and treatment algorithms guidelines of the NTLP and NTRL governed the use of smear microscopy and Xpert at participating health centers. At the time of this study, Uganda national guidelines called for Xpert testing in persons living with HIV, health care workers, contacts of drug-resistant (DR-TB) patients, pregnant women or breast-feeding mothers, prisoners, patients from refugee camps, and diabetics.

### Study design

This cross-sectional study combines several sources of triangulated quantitative and qualitative data to assess the coverage, penetration, and fidelity to implementation of Xpert testing in Uganda’s hub and spoke model from the perspective of level III-IV community health facilities.

### Data collection

Data for this study was obtained in three ways: surveys, data abstraction from routine health registers, and field notes recorded by study staff from observing community health center staff, work processes and personal communication. This data was collected as part of a mapping exercise in preparation for studies conducted by the Uganda Tuberculosis Implementation Research Consortium (U-TIRC) aiming to assess and improve TB diagnostic evaluation in Uganda.

First, to assess characteristics of the community health centers, a research staff member administered a site assessment survey to clinic staff using a standardized tool (Supplement file) developed for this study to capture information on infection control procedures; laboratory procedures; TB testing and treatment procedures; and utilities, infrastructure and resources. Survey participants included (in order of priority based on seniority, in keeping with norms in Uganda) any of the following individuals available at the time of the study staff site visit: the health center director (also known as the facility in-charge), laboratory director, and/or TB focal person at each health center.

Second, to obtain patient-level data on utilization and outcomes of Xpert testing, we reviewed routine TB registers including NTLP laboratory registers, Xpert referral forms, an electronic reporting platform for GeneXpert results (GxAlert, SystemOne Northampton USA) and NTLP treatment registers, all for the period January – December 2016. We included data on all patients undergoing TB testing who had indications for Xpert testing per Uganda NTLP guidelines at the time the survey was completed. To ensure that we captured Xpert testing results, we also downloaded testing data directly from the Xpert devices at testing hubs serving each participating community health facility. We developed a standardized data extraction tool to match patients across data sources. Data abstracted included age, sex, date of initial sputum collection, method of sputum examination (smear microscopy, Xpert), sputum examination results, date of results, date of treatment initiation, and HIV status. All the data abstraction, collection, and entry was done by trained research assistants using secure mobile-based Research Electronic Data Capture (REDCap, Nashville USA) [7] platform hosted by the University of California San Francisco. To ensure data quality, data was subjected to a rigorous quality assurance process using REDCap generated reports on a bi-monthly basis by our data management team.

Finally, qualitative data was collected from field notes taken by study staff during site visits. Staff recorded observations about the TB diagnostic evaluation process in participating community health centers during site visits for trainings, surveys, and data abstraction. Staff were asked to take notes related to the process of specimen collection, specimen transport, specimen testing, result reporting and patient linkage to treatment initiation if diagnosed with TB.

### Data analysis

**Fig. 1:**
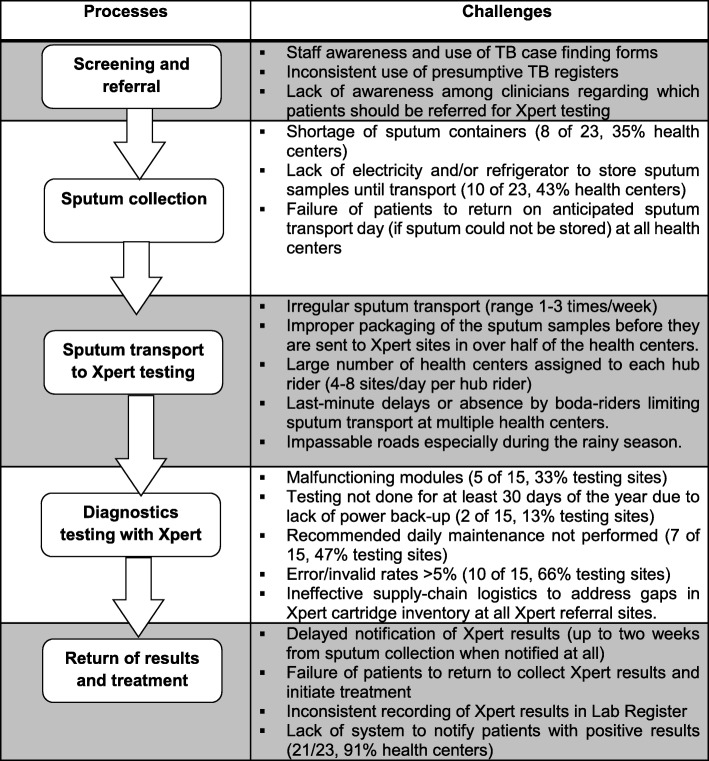
Health systems challenges to GeneXpert implementation during the TB diagnostic evaluation cascade of care. Challenges to TB diagnostic evaluation at community health centers synthesized from both quantitative and qualitative data are mapped to specific steps in the diagnostic evaluation process

We compared differences in proportions across sites using the χ^2^ test of proportions. Qualitative data from field notes were organized by health center, transcribed and reviewed for thematic interpretation using a framework analysis to identify key issues related to the hub-and-spoke model of TB diagnostic evaluation. We used qualitative inquiry to elucidate site specific and general health system barriers to Xpert testing. While formal coding was not conducted, data were summarized into categories corresponding to processes of care for TB diagnosis (Fig. 1). We performed all quantitative analyses using Stata version 14 (Stata Corporation, College Station, TX). Qualitative data was categorized by theme and reviewed in Excel (Microsoft, Redmond, WA).

The study was approved by the School of Medicine Research and Ethics Committee at Makerere University, the Uganda National Council for Science and Technology, and the University of California San Francisco Committee on Human Research.

## Results

### Site assessment survey

#### Health center characteristics

Of the 23 participating community health centers, 6 (26%) were in the Eastern Region of Uganda and 17 (74%) in the Central Region. Health centers were classified as Level IV (*n* = 15, 65%) or Level III (*n* = 8, 35%), and all provided on-site sputum smear microscopy services. Enrolled health centers were often located in rural (*n* = 14, 61%) areas and at a distance of approximately 21.5 km (km) (IQR 13–34.5) from the closest Xpert testing hub.

Physical infrastructure, availability of water and sanitation facilities, and availability and consistency of electricity varied across the health centers (Table 1). Twenty-one sites (91%) had consistent electricity and 22 (92%) had functioning water and sanitation systems, however only 13 (57%) reported having refrigerators in their laboratories. Of the 14 rural health centers, a notable proportion harvested rain water as their primary water source (*n* = 5, 36%) and relied on solar panels for electricity (*n* = 6, 43%), although only critical departments such as the operating theatre and the laboratory were connected to solar power. Communication infrastructure and capability was also limited in a majority or health centers. Of the 23 community health centers, only 2 (9%) had access to and sufficient airtime to use mobile phones to communicate with testing facilities or patients.

**Table 1:** Characteristics of participating community health centers and Xpert hubs based on health center survey and field notes

Health Center Characteristics	Proportion or Median (IQR) (*N* = 23)
HC IV	65% (15)
Rural	61% (14)
Distance to nearest Xpert testing facility (km)	20.3 (13–34.5)
Have consistent electricity	91% (21)
Refrigerator	57% (13)
Have functioning water/sanitation system	92% (22)
Number of health workers	20 (15.5–25)
Transportation (weekly)	43% (10)
Notification of positive results to patients (phone call)	13%(2)
Number of Xpert tests done	13.5 (11–20)

#### Health center staffing

In accordance with Ministry of Health staffing requirements, Level III health centers were led by a senior clinical officer holding a diploma in clinical medicine, who ran the general outpatient department (OPD), whereas the Level IV health centers were led by a senior medical officer holding a degree in medicine. In addition, only Level IV health centers had a designated TB focal person responsible for managing the TB unit and TB treatment register, and a laboratory technologist responsible for supervising laboratory technicians. At both Level III and IV health centers, staff rotated daily with some assigned to work at least 2–3 days per week and others working full time from 9 am to 5 pm.

### Chart abstraction

#### GeneXpert utilization

During the study period, of the 2747 adults with presumed TB, 2192 (79.8, 95% CI 78.2–81.3%) were referred for TB testing. Of the 1597 (72.9 95% CI 70.9–74.7%) patients who were eligible for Xpert testing based on NTLP guidelines, only 574 (35.9, 95% CI 33.6–38.4%) had sputum referred for Xpert testing. Patients referred for Xpert testing included 76 smear-negative patients (13.2 95% CI 10.6–16.3%) and 481 persons living with HIV regardless of smear status (83.8 95% CI 80.5–86.7%). Xpert was requested as the first line test for only 39 of 2192 (1.8, 95% CI 1.3–2.4%) patients. Of the 54 patients confirmed to have TB by Xpert testing, 33 (61.1, 95% CI 46.9–74.1%) were initiated on TB treatment overall and 25 (46.3, 95% CI 32.6–60.4%) within 14 days. Median time to treatment was longer for patients confirmed to have TB by Xpert testing (2 days, IQR 0–14) than for those confirmed by smear microscopy (0 days, IQR 0–1). The cumulative proportion of patients diagnosed with TB who started on treatment was lower for patients confirmed to have TB by Xpert testing than for those confirmed by smear microscopy at both 7 days (38.9, 95% CI 25.9–53.1% vs. 80.5, 95% CI 73.7–86.2%, *p* < 0.001) and 14 days (46.3, 95% CI 32.6–60.4%, vs. 95, 95% CI 80.3–91.2%), *p* < 0. 001). Only one patient was identified as having rifampin (RIF) resistant TB. Even when patients obtained Xpert testing, 15% of the Xpert testing hubs experienced at least one non-functional module with error/invalid rates greater than 5% at 10 (67%) testing hubs.

### Field notes

#### Specimen collection

Sputum samples that were collected to be sent for Xpert testing were packaged in specialized sputum mugs with a tight lid to avoid leakage. Of 23 sites, 8 (35%) experienced shortages of these sputum mugs, and 10 (43%) lacked electricity to enable refrigerated storage of samples until they were transported for testing (Fig. 1). Sites that lacked refrigeration equipment noted that patients who were asked to return for sputum collection on scheduled sputum transport days frequently failed to come even when asked to do so.

#### Specimen transport

Sputum samples collected from patients were transported to Xpert testing hubs 1–3 times a week, with many of the health centers (*n* = 10, 43%) transporting specimens at least once a week (Fig. 1). All health centers used a *boda* rider to transport sputum samples to Xpert referral sites, with each boda rider allocated 4–8 health centers per day. Delays of greater than 3 days in transporting sputum samples were noted at 5/23 (22%) health centers.

#### Specimen testing

At the Xpert testing hubs, the numbers of samples received are recorded in the Xpert site laboratory register as they are tested. A lack of electricity prevented testing from being performed on at least 13% of the working days year at Xpert testing hubs because of lack of electricity (Fig. 1). Standard recommended daily maintenance was not performed at 7/15 (47%) testing sites on any day of the year. Ten of 15 (66%) Xpert testing sites recorded error/invalid rates > 5%, which exceeds quality control limits. Finally, all Xpert testing sites reported unreliable logistics for the ordering and delivery of Xpert cartridges to maintain local site inventories.

#### Results receipt and notification

Positive Xpert results were variably communicated back to health centers (Fig. 1). Some Xpert sites communicated through a phone call or text message (SMS) to referring health centers, while other hub-and spoke dyads relied on when *boda* rider’s return to the referring health center to disseminate test results. Results for patients referred from each health center were printed from the Xpert machine when the corresponding *boda* rider came to the Xpert site in order to verify that the specific results provided matched the identifiers for all of the samples that were delivered. After confirmation, the *boda* rider returned the results to the facilities on the next scheduled sputum pick-up day. A majority of the sites experienced delays in notification of Xpert results of up to 2 weeks after the initial request. In some cases, results were returned only after patients had returned for them at the sites. At the health centers, staff inconsistently recorded Xpert results in the TB laboratory register. Study staff noted that when asked, a majority of health center staff including TB focal persons were not able to identify or characterize critical process metrics for TB care in their clinic (Fig. 1). TB focal persons at each community health center attempted to contact patients who provided a phone number, asking them to return to the health center to receive their Xpert test results and initiate treatment as appropriate. Patients who did not have a telephone number recorded could not be contacted and the facility had to wait until such a patient returned to the health center to initiate treatment. The cumulative delays in reporting and recording Xpert test results were acknowledged to cause delayed treatment initiation for patients who were microbiologically confirmed to have TB (Fig. 1).

## Discussion

Our study confirms that Xpert utilization was poor among community health centers in Uganda linked to Xpert testing hubs. Only 35.9% (95% CI 33.6–38.4%) of patients eligible for TB testing were referred for Xpert testing and only 1.8, 95% CI 1.3–2.4%) of patients with presumed TB received Xpert as a first line test. This is despite national policy guidelines which recommend universal testing with Xpert when linkage to Xpert testing is available, and specifically for people living with HIV. Even when patients with presumed TB underwent Xpert testing the median time to treatment initiation was longer than for those who were diagnosed with smear microscopy (median 2 days IQR 0–14 for Xpert compared to median 0 IQR 0,1 for smear microscopy). This result suggests that current systems for patient linkage to Xpert have failed to improve the efficiency of TB testing. Together these data suggest that current methods for decentralizing Xpert testing using a hub-and spoke model for decentralization fail to increase both uptake of Xpert testing and improvements in process metrics such as time to treatment initiation for TB patients, thereby contributing to the low impact of Xpert in high-burden settings.

Our findings are in line with other data from Uganda reporting overall low utilization rates for Xpert testing, with only about 20% of eligible patients at community health centers receiving Xpert, and less than 2% being referred for Xpert testing as a first-line test [8, 9]. A study in Malawi evaluating the impact of Xpert roll-out on TB diagnosis and treatment showed only a small contribution to case detection. This low diagnostic impact was attributed to operational challenges and other gaps in the continuum of care such as difficulty in sputum transportation and slow turnaround time in high volume settings [10]. Results from our surveys of community health centers and from field notes reveal additional detail regarding operational challenges and gaps in the TB diagnostic cascade of care that impact Xpert effectiveness. These included challenges at community health centers that prevent collection and transport of sputum (stock-outs of sputum collection supplies). Limitations with sputum transportation were documented, due to an overextended motorcycle rider network with ad hoc back-up systems. Xpert testing sites, had non-functioning modules for extended periods of time and high error/invalid rates, making testing ineffective. Finally, communication of results to health centers and patients was limited (91% of health centers could not relay positive Xpert results because of a lack of a mobile phone airtime). When taken in aggregate, our study documented serious systems issues that negatively impact the entire process of referral-based Xpert testing. In addition, the variability of system performance, capacity and efficiency at these health centers suggest that more central coordination is required to support decentralized Xpert testing platforms.

Our results reflect detailed data collection from health centers in 15 districts of Uganda spanning all 4 administrative regions of the country. This sampling strategy suggests that our results may be generalized to rural and peri-urban TB care across the country. Our ability to identify challenges at each level in the Xpert referral implementation process aligns with the patient cascade of TB diagnostic care in Uganda [11], which demonstrates significant of pre-treatment loss to follow-up within this context. Our approach in understanding operational challenges to Xpert hub-and-spoke implementation may inform the development of targeted strategies to address these challenges while improving patient outcomes.

Although our results reflect detailed data collection from health centers in 15 districts of Uganda spanning all 4 administrative regions of the country, our study had some limitations. We were unable to collect data on gaps in health center resources and supply systems as health center staff were unable to document or characterize supply chain operations and resource availability. Health workers in some instances were unable to describe or document standardized operating procedures for the process of referring samples for Xpert testing and reporting results. However, these data lapses reinforce our results suggesting the limited capacity of facility staff and providers to facilitate high-quality Xpert testing services. Additionally, while we obtained perspectives from front-line staff regarding gaps to provision of Xpert testing locally, we did not obtain additional perspectives from NTRL staff nor did we attempt to conduct a quantitative analysis of operational gaps at the national level.

Our results suggest the need for re-conceptualizing what is meant by TB diagnostic “capacity” at the health system level beyond simply increasing numbers of commodities, such as Xpert devices. A more horizontal health systems approach to support integration, sustainability, and reach of this technology is required to improve implementation of Xpert and enhance its potential effect on TB prevention and care. Funders who have been supporting scale up of Xpert as well as other technologies to improve TB prevention and care should consider supporting systems that optimize the functionality of Xpert devices and not only focusing on procurement. Monitoring and evaluation of Xpert implementation could be improved by adding monitoring of facility resources, infrastructure, and personnel capacity to carry out Xpert testing, including coordination or referral and reporting in addition to device specific quality assurance [12]. Our results suggest that integration and sustainability of Xpert testing across the health system, could be improved with better improved and standardized systems bi-directional communication and linkage between testing facilities and referring health centers, on-going training and support for providers to maintain networks, and programmatic provisions to support the maintenance and use of Xpert technology. Strategies that incorporate novel laboratory services within existing health and laboratory service delivery schemes have the benefit of strengthening health systems while achieving disease specific goals [13].

## Conclusions

This study highlights the need for a more holistic health systems approach to the deployment and integration of technologies such as GeneXpert in routine health settings. Additional operational, implementation, and applied health systems research to design robust these strategies is a first step in maximizing the public health impact of these of novel interventions.

## Supplementary information

**Additional file 1.** Site Assessment Tool. A survey tool used to systematically collect information about the infrastructure and capacity for tuberculosis diagnostic testing at participating community health centers.

## Data Availability

Datasets generated, used and analyzed in this study are available from the corresponding author upon reasonable request.

## Abbreviations

CI: Confidence Interval
CPHL: Central Public Health Laboratories
DR-TB: Drug Resistant Tuberculosis
HIV: Human Immunodeficiency Virus
IQR: Interquartile Range
NTLP: National Tuberculosis and Leprosy Programme
NTRL: National Tuberculosis Reference Laboratory
OPD: Outpatient Department
RIF: Rifampin
SD: Standard Deviation
SMS: Short Message Service
TB: Tuberculosis
UCSF: University of California San Francisco
U-TIRC: Uganda Tuberculosis Implementation Research Consortium
