# Supplementary material for: Challenges with scale-up of GeneXpert MTB/RIF® in Uganda: a health systems perspective
Source: BMC Health Serv Res. 2020 Mar 4;20:162. doi: 10.1186/s12913-020-4997-x (PMC7057496; doi:10.1186/s12913-020-4997-x)
Supplement: Supplementary file 1 — Additional file 1. Site Assessment Tool. A survey tool used to systematically collect information about the infrastructure and capacity for tuberculosis diagnostic testing at participating community health centers. [file 12913_2020_4997_MOESM1_ESM.docx]

Site:

Date:

Study Staff Conducting Assessment:

**ADMINISTRATION**

| **Health Centre leaders** | | **Name** | **Contact** | **Comments** |
| --- | --- | --- | --- | --- |
| 1 | Health Centre in-charge |  |  |  |
| 2 | Laboratory in-charge |  |  |  |
| 3 | TB site focal person |  |  |  |
| 4 | TB drug dispenser |  |  |  |
| 5 | Records officer |  |  |  |

| **TB screening/infection control procedures** | | **Response** | **Comments** |
| --- | --- | --- | --- |
| 1 | Is any screening for TB done when patients are registered or in the waiting area? If yes, specify |  |  |
| 2 | Is any health education related to TB provided in the waiting area? If yes, specify and indicate whether TB suspects are asked to self-identify for testing. |  |  |
| 3 | Is there an HIV clinic at the facility? If yes, is TB screening done regularly at the HIV clinic? |  |  |

| **TB TESTING/TREATMENT PROCEDURES** | | **Comments** |
| --- | --- | --- |
| 1 | Which patients are sent for TB testing? |  |
| 2 | Where is TB treatment provided ie TB/HIV clinic, dispensary, TB clinic |  |
| 3 | Are anti TB drugs regular/ Does the facility always have anti TB drugs |  |

| **LABORATORY PROCEDURES** | | **Comments** |
| --- | --- | --- |
| 1 | What type of microscopy is performed (light or LED FM)? Is the microscope functioning? |  |
| 2 | In the past one year, has there been a regular supply of TB microscopy related materials (sputum cups, staining materials, slides)? |  |
| 3 | Are samples being referred for Xpert testing? For which patients? |  |
| 4 | How are samples transported to the Xpert site and how often? |  |
| 5 | How long does it take to get Xpert results back? |  |
| 6 | Are there any plans to obtain an Xpert machine for the facility? |  |

| **TB REGISTER DATA** | | **Number** | **Comments** |
| --- | --- | --- | --- |
| 1 | Number of patients tested for TB by microscopy in the past one year (check the lab register) |  |  |
| 2 | Number of smear-positive TB cases diagnosed at site in last year? (check lab register) |  |  |
| 3 | Number of GeneXpert referrals made in last year. Number of patients diagnosed with TB by GeneXpert. (per lab register or estimate of lab in-charge) |  |  |
| 4 | Number of new TB cases started on treatment in the past one year (check treatment register) |  |  |

| **ASSESSMENT OF UTILITIES** | | **Response** | **Comments** |
| --- | --- | --- | --- |
| 1 | Are there power outages in the clinic? |  |  |
| 2 | If so, how often (days/month) and how long do they last? |  |  |
| 4 | Is there back up power in the lab? If yes, what kind and does it function well? |  |  |
| 7 | Is the water supply regular? |  |  |
| 10 | Does the clinic/lab have a telephone? How is it used? |  |  |
| 11 | Does the clinic have a motor bike or car for patient follow-up? Is it being used? |  |  |

| **SUPPORT/OTHER PROJECTS** | |  |
| --- | --- | --- |
| 1 | Are there projects that focus on TB at the facility? |  |
| 2 | What is their focus? Specify |  |
| 3 | What kind of support do they give? |  |
| 4 | Are there project specific Patient Record Forms and for which patients are they used? |  |
| 5 | Are there TB specific trainings or refresher courses that staff participate in? If so, how often and what type? |  |

| **SECURITY AT THE FACILITY** | | **Comments** |
| --- | --- | --- |
| 1 | Have you had any security incidents such as theft or property damage to the clinic this in the past year? |  |
| 2 | Doors i.e. lab, pharmacy and records |  |
| 3 | Burglar proof Windows |  |
